# The Dimensionality of the 12-Item General Health Questionnaire (GHQ-12): Comparisons of Factor Structures and Invariance Across Samples and Time

**DOI:** 10.3389/fpsyg.2020.01300

**Published:** 2020-06-11

**Authors:** Sigurd W. Hystad, Bjørn Helge Johnsen

**Affiliations:** Department of Psychosocial Science, University of Bergen, Bergen, Norway

**Keywords:** General Health Questionnaire (GHQ-12), confirmatory factor analysis (CFA), bifactor models, wording effects, factor structure, measurement invariance, military

## Abstract

Because of its brevity, the 12-item General Health Questionnaire (GHQ-12) has become one of the most popular and used measure for detecting psychological distress. Originally intended as a unidimensional measure, the majority of subsequent factor-analytic studies have failed to support GHQ-12 as a unitary construct and have instead proposed a plethora of multidimensional structures. In this study, we further examined the factor structure in two different military samples, one consisting of crewmembers from four different frigates deployed in anti-piracy operations and Standing NATO Maritime Group deployments (*N* = 591) and one consisting of crewmember from three different minehunters/sweepers serving in Standing NATO Mine Counter-Measures Group deployments (*N* = 196). Results from confirmatory factor analyses (CFA) performed in the first sample supported a bifactor model, consisting of a general factor representing communality among all items and two specific factors reflecting common variance due to wording effects (negatively and positively phrased items). A multi-group CFA further confirmed this structure to be invariant across our second sample. Structural equation modeling also showed that the general factor was strongly associated with symptoms of insomnia and mental health, whereas the specific factors were either non-significantly or considerably weaker associated with the criterion variables. Overall, our results are congruent with the notion that the multidimensionality demonstrated in many previous investigations is most likely an expression of method-specific variance caused by item wording. The explained unique variance associated with these specific factors was further relatively small. Ignoring the multidimensionality and treating GHQ-12 as a unitary construct will therefore most likely introduce minimal bias to most practical applications.

## Introduction

Increased focus on mental health problems and its impact on the population have resulted in development of screening programs for different sub-groups at risk for developing severe psychopathologies. The diversity in programs range from screening for mental health in women with risk of transferring HIV to their children (Iheanacho et al., 2015) to mental health evaluation of elderlies in Korean communities (Ju et al., 2017). Mental health screening in the military domain is parallel to community screening programs and has long historical roots (Wright et al., 2002). At present, most nations implement programs before and after deployments to operational areas (Rona et al., 2005), and some nations have an additional mid-operation evaluation (Sanden et al., 2014). The main aims of these screening procedures are early case identification in order to implement adequate interventions as well as defining specific stressors for the personnel involved and for the types of missions in which they are involved. In order to be able to process large amount of data in brief periods of time, short self-report inventories are preferable. Shorter inventories do not only reduce the assessment time and related costs but can also improve participation rates and reduce participation fatigue, and as such lead to better data quality. However, many researchers have also pointed out the inevitable trade-off between these pragmatic reasons and the psychometric quality that is lost when using shorter or abbreviated scales (Smith et al., 2000; Credé et al., 2012). For instance, Credé et al. (2012) reported lower internal consistency estimates and lower predictive power across a range of outcomes for short versus long scales.

One short questionnaire often used in both community and military screening is the 12-item version of the General Health Questionnaire (GHQ-12; Goldberg and Williams, 1988). The GHQ-12 derives from the original 60-item version, and additionally exists in 30-, 28-, and 20-items versions (Goldberg and Williams, 1988). The advantage of GHQ-12 is that it is short, can easily be scored “clinically” (symptoms present or absent) as well as levels of symptoms present (Likert-type scoring). The scale was originally designed as a screen for risk for common mental disorders (Böhnke and Croudace, 2016), but has also been used as a measure of general symptom load (Johnsen et al., 1998), Positive mental health (Hu et al., 2007) and minor psychological problems (Nordmo et al., 2020). The instrument is frequently used in screening of civilian populations in different cultures (Iheanacho et al., 2015; Endsley et al., 2017; Ju et al., 2017; Tseliou et al., 2018). The frequent use of the scale in different cultures and the different interpretations of what the scale measures, motivates for a psychometric analysis in order to clarify the validity of the instrument.

### Dimensionality of GHQ

The GHQ-12 was intended as a unidimensional measure of psychological distress (Goldberg and Williams, 1988). Because of its brevity, the GHQ-12 has become one of the most used instruments for detecting psychological distress in non-clinical samples (Hankins, 2008; Tomás et al., 2017). The instrument has been translated into many different languages, including Spanish (Cuéllar-Flores et al., 2014), Portuguese (Tomás et al., 2017), German (Romppel et al., 2013), French (Salama-Younes et al., 2009), Italian (Politi et al., 1994), Dutch (Cornelius et al., 2013), Norwegian (Nordmo et al., 2020), Farsi (Namjoo et al., 2017), Japanese (Suzuki et al., 2011), Thai (Gelaye et al., 2015), and Chinese (Ye, 2009). Although most research to date has used the GHQ-12 to compute a global distress score, the structure and dimensionality of the measure is still a matter of debate. In fact, the most common finding from the many studies that have explored the dimensionality of GHQ-12 is the failure to find support for a single-factor structure.

The most common alternatives emerging from exploratory analyses seem to be either a two-factor or a three-factor structure. In one early study, Politi et al. (1994) identified two different factors that they labeled “General dysphoria” and “Social dysfunction,” which they also found to have differing discriminatory power. Similar two-factor structures have been found in several later and more recent studies, although the nomenclature and qualitative meaning designated to the different factors have varied somewhat across studies. For example, Centofanti et al. (2019) considered the second factor to be an expression of “General functioning” rather than “Social dysfunction,” while Glozah and Pevalin (2015) considered the first factor to reflect “Social anxiety” rather than “General dysphoria.” Others have used labels such as “Anxiety/Depression,” “Distress” and “Loss of positive emotions” (Doi and Minowa, 2003; Sarková et al., 2006; Suzuki et al., 2011; Gao et al., 2012). Adding to the confusion are studies that present qualitatively similar factors, but which often contain noticeably different factor loading patterns (e.g., Montazeri et al., 2003; Cuéllar-Flores et al., 2014; Gelaye et al., 2015).

Alternative models with three factors also exist in the literature (Picardi et al., 2001; Doi and Minowa, 2003; Padrón et al., 2012; Gelaye et al., 2015; Guan, 2017), of which the model proposed by Graetz (1991) have gained the most attention and have later been replicated in confirmatory analyses (French and Tait, 2004; Shevlin and Adamson, 2005; Abubakar and Fischer, 2012). This model distinguishes between “Anxiety,” “Social dysfunction” and “Loss of confidence.” The social dysfunction factor in Graetz’s model mirrors the namesake factor in Politi et al. (1994) model, whereas the anxiety and loss of confidence factors is a breakdown of the general dysphoria factor into two different factors.

A number of two- and three-factor models have routinely also been tested within a confirmatory factor-analytical framework, with support found for both types of models. Several studies have found a two-dimensional representation to fit the observed data best, but these are not always comparable as they differ in respect to the latent factor content, the parameterization of the model or even the number of items included in the analysis. For example, studies have found support for two factors based on a reduced 7-item (Wong and O’Driscoll, 2016), 8-item (Kalliath et al., 2004; Ip and Martin, 2006), or 10-item (Salama-Younes et al., 2009) version of the GHQ. Others have included correlations between the unique variance of items without providing any logical or theoretical justifications for these additions (e.g., Namjoo et al., 2017). Confirmatory three-factor models, in contrast, have for the most part followed the model proposed by Graetz (1991). For example, French and Tait (2004), Shevlin and Adamson (2005), and Abubakar and Fischer (2012) all found the three-factor model to be the best fitting model among those tested (which also included a two-dimensional model).

A major problem with both the dominating two-dimensional model by Politi et al. (1994) and the three-dimensional model by Graetz (1991) is the separation of negatively and positively phrased items into separate factors. The GHQ-12 consists of an equal number of positively and negatively phrased items, and it is well known that when psychological rating scales contain a mix of negatively and positively phrased items, factor analyses of these items often reveal apparently distinct factors reflecting the wording of the items (Marsh, 1996). This is indeed the case with both the two-factor and three-factor models. In Politi et al. (1994) two-factor structure, all positively worded items loaded on one factor and all negatively phrased items loaded on the other factor. The only exception was item 12 (“Been feeling reasonably happy”), which loaded about equally on both factors. Similarly, in Graetz’s three-factor model, one factor contains all the positively phrased items, while the negatively phrased items are divided into two separate factors. In cases like these, the question arises whether these factors are substantively meaningful factors or artifacts of response styles associated with the positively and negatively phrased items.

In response to this challenge, later studies have explicitly tried to model wording effects in confirmatory factor models. Hankins (2008) compared a two- and three-dimensional model with a unidimensional model that incorporated wording effects by allowing correlated error terms on the negatively phrased items. Results from this comparison demonstrated that the unidimensional model with wording effects provided a better fit than both the two-dimensional and three-dimensional model. While correlated errors are clearly indicative of systematic error variance, they do not necessarily point to a single, common method factor as an explanation, as several different latent factors may cause these correlations. However, Ye (2009) took a similar approach and modeled a specific method factor associated with the negative items in addition to a general factor representing general distress. Ye found that this model provided a good fit to the data, although both a two- and three-dimensional model fitted equally well.

Studies have extended this logic by including two separate specific factors, one for the negatively phrased items and one for the positively phrased items. This sort of model is often referred to as a bifactor model, and are used in situations when the covariance among a set of items can be accounted for by a single, unidimensional factor that represents the communality among all the items, in addition to domain-specific factors that reflect additional common variance among subsets of the items (Reise et al., 2007). Bifactor modeling has some advantages over traditional factor models, because it allows us to examine if a measure is essentially unidimensional or if the items are multidimensional and whether subscale scores provide additional reliable information beyond the total score (Reise et al., 2007, 2013a). For example, in addition to traditional fit statistics, bifactor models also offers the opportunity to evaluate the percentage of common variance that can be attributed to the general factor in the model (Reise et al., 2013b).

Both Tomás et al. (2017) and Centofanti et al. (2019) included bifactor models among the different factor structures that they tested, with somewhat mixed results. Centofanti et al. (2019) found that the bifactor was the best fitting model and reported an omega hierarchical (ω_h_) value of 0.81. Omega hierarchical is an expression of the total amount of observed score variance that is attributable to the general factor in a bifactor model, and ω_h_ = 0.81 thus supports the presence of a strong general distress factor. Tomás et al. (2017), on the other hand, found that a bifactor model did not improve the fit over a three-factor model based on Graetz (1991).

### Aims of the Current Study

The importance of valid and easy to use tools for screening military personnel cannot be overestimated. For instance, the scale of United States military deployments is extremely high with 7.5 million troops deployed since 9/11 (McCarthy, 2018). As an example of a European nation, the United Kingdom has deployed almost 300,000 troops to Afghanistan and Iraq alone (Ministry of Defence, 2015). Norway has over the last decades increased their participants in international operations and it is estimated that about 100,000 soldiers have been deployed to 40 countries since world war two (Norwegian Armed Forces, n.d.).

The widespread use of the GHQ-12 both in civilian and military settings combined with the ongoing uncertainty regarding its factor structure, motivated us to scrutinize further the psychometric properties of the measure. Despite the many different models that have been proposed and tested in the literature, there is still no consensus regarding the most appropriate dimensional description of the GHQ-12. Because the factor structure of the GHQ-12 to a large degree seems to vary from study to study and sample to sample, it is also important to examine whether the factor model identified as the best structure can be replicated in different samples or over time in the same sample. Testing for measurement invariance of the GHQ-12 allows us to examine whether the items of the overall distress factor or any sub-factors are interpreted the same across samples and measurement points.

If the GHQ-12 measures qualitatively different constructs rather than a general and unidimensional mental health factor, then we should expect the different factors also to have distinct nomological networks. Furthermore, if the subscales are to offer any utility, then a multidimensional model should offer unique predictive validity beyond a general GHQ-12 factor, in addition to providing a statistically better fit in a confirmatory factor analysis. Shevlin and Adamson (2005), for example, questioned the utility of the three-factor model they found to be the best representation of the data, because the three factors provided little information beyond that of a general factor. In the present paper, we plan to examine the associations between GHQ and symptoms of insomnia and mental health.

The aims of this article were therefore to (a) test and compare the different models that have been proposed in previous studies; (b) assess whether the model identified as the best fitting model was invariant across samples and across time; and (c) explore whether the different latent factors underlying the GHQ-12 (if any) have distinct nomological networks or predictive validity beyond a general factor.

## Materials and Methods

### Samples

A total of 591 crewmembers from four different frigates serving in the Royal Norwegian Navy comprised our first sample. The frigates were deployed in international anti-piracy operations and standing NATO maritime deployments at various periods during 2013–2017. This sample served as our principal sample that we used to test and compare the various factor models, as well as explore the predictive utility and test the invariance of the best fitting model across two time-points.

Crewmembers (*N* = 196) from three different minehunters/sweepers serving in standing NATO mine countermeasure group deployments (between 2014 and 2017) served as the second sample. This sample was used to test if the factor model identified as the best fitting model in Sample 1 was invariant across groups. Both samples belonged to vessels sailing in international operations. Normal deployment cycles are 6 and 4 months for frigates and mine-countermeasure vessels, respectively.

The above samples were chosen because although both military, there are also some key differences between them. Compared with minehunters, frigates are larger vessels and are usually manned by a crew of about 120 sailors. Minehunters are considerably smaller, with a crew of about 35. The crew onboard frigates are on average older and comprises relatively more officers and enlisted personnel. The management structure onboard also differs. Due to their size, frigates are characterized by a stronger hierarchical structure, which further entails that the leadership is less direct and to a larger degree executed through department heads. Minehunters, on the contrary, are characterized by less social distance and a more direct leadership structure. Frigates further have the capacity for longer times at sea without replenishment and have more varied operational capacities, including green-water (coastal) and blue-water (open ocean) operations. Minehunters, on the other hand, are less self-sufficient and generally spend less time at sea, and their operational capacities are more weather dependent and restricted to coastal waters.

### Procedure

The data collection was part of the standard procedure for psychological evaluation in the Royal Norwegian Navy (see Sanden et al., 2014, for an overview). The procedures include pre-deployment screening as well as mid- and post-deployment evaluation. The post-deployment screening was conducted while transiting back to the Norwegian home base. In the current paper, we use data from the pre- and post-deployment screenings.

### Measures

#### General Health Questionnaire-12 (GHQ-12)

The GHQ-12 consists of 12 statements to which respondents indicate agreement on a four-point scale (0 = *Not at all*; 3 = *More than usual*; Goldberg and Williams, 1988). All items are available in Table 2.

#### Bergen Insomnia Scale (BIS)

The BIS consists of six items measuring different aspects of insomnia (e.g., sleep onset, early morning wakening and daytime impairment), constructed based on the inclusion criteria for insomnia in the *Diagnostic and Statistical Manual of Mental Disorders* (Pallesen et al., 2008). For each item, participants indicate how many days per week during the last month they experienced problems with that particular aspect of sleep. Each item is rated on an 8-point scale, ranging from 0 to 7 days per week. The items can be combined to create a single insomnia score. An example item is: “During the past month, how many days a week has it taken you more than 30 min to fall asleep after the light was switched off?” (sleep onset).

#### Hopkins Symptom Checklist-25 (HSCL-25)

The HSCL-25 is a 25-item screening tool designed to detect symptoms of anxiety and depression (Derogatis et al., 1974). Respondents are asked to indicate to what degree (1 = *not at all*; 4 = *very much*) each of the 25 symptoms have been troubling or concerning them during the last 2 weeks. Example items are “Suddenly scared for no reason” and “Spells of terror or panic.” All items can be combined to form a total distress score. Alternatively, the first 10 items can be used to create an anxiety score and the last 15 items can be used to create a depression score.

### Statistical Analyses

**FIGURE 1:**
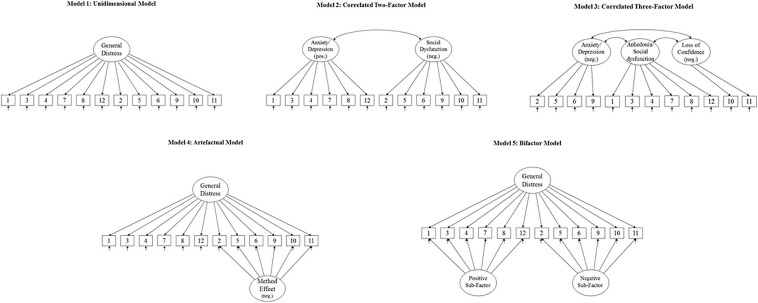
The different factor models for GHQ-12 tested and compared.

We planned to examine a range of different factor models previously used in the literature, illustrated in Figure 1 and briefly described below:

#### Model 1

A unidimensional model with a single factor explaining the covariance between all items.

#### Model 2

A model with two correlated latent factors containing one factor with all negatively phrased items and one factor with all positively phrased items. This model was originally proposed by Andrich and van Schoubroeck (1989) and is identical to the General dysphoria and Social dysfunction factors proposed by Politi et al. (1994), except for one item that loaded on both factors in the latter study. For conceptual clarity, we do not include this double loading.

#### Model 3

A correlated three-factor model originally suggested by Graetz (1991). The three latent factors in this model represent “Anhedonia/Social dysfunction” (all positively phrased items), “Anxiety/Depression” (four negatively phrased items) and “Loss of confidence” (two negatively phrased items). The major difference between this model and the previous correlated two-factor model is that it divides the negatively phrased items into two distinct latent factors.

#### Model 4

A unidimensional model with an additional orthogonal method factor specifically for the negative items (Ye, 2009). This model reframes Model 2 as an artifactual division into different factors caused entirely by wording effects.

#### Model 5

This model extends Model 4 to include an orthogonal specific factor for the positively phrased items as well. This type of model is often referred to as a bifactor model (Reise et al., 2007) and has previously been tested by Tomás et al. (2017) and Centofanti et al. (2019).

#### Model Fit and -Comparison

Individual model fit was evaluated by examining the size and statistical significance of factor loadings, as well as several commonly used goodness-of-fit statistics. Specifically, we used the comparative fit index (CFI), the standardized root mean square residual (SRMR) and the root mean square error of approximation (RMSEA), together with its 90% confidence interval (CI). MacCallum et al. (1996) have suggested that values of 0.01, 0.05, and 0.08 for RMSEA correspond to excellent, good and mediocre fit, whereas a value less than 0.08 for SRMR is generally considered a good fit (Hu and Bentler, 1999). For CFI, a value close to 0.95 indicates a good fit between the hypothesized model and the observed data, whereas values in the range of 0.90–0.95 are considered acceptable (Kline, 1998; Hu and Bentler, 1999; McDonald and Ho, 2002).

To compare the competing models, we used two measures of comparative fit, The Akaike information criterion (AIC) and the Bayesian information criterion (BIC), as well as the likelihood-ratio test when appropriate. Lower values for both AIC and BIC indicate a better fit.

#### Predictive Validity

The factor model identified as the best fitting model was included in a full structural model (SEM) with insomnia (BIS) and mental health problems (HSCL-25) as endogenous latent variables. We formed item parcels of the indicators for both BIS and HSCL in order to keep the complexity of the model to a minimum. When the interest lies in the structural relationships rather than the measurement parameters, items parceling can be defensible under some preconditions (Bandalos and Finney, 2001). Importantly, items should be combined only within unidimensional domains. Previous factor analyses of BIS have suggested both a single factor and a two-factor solution where nocturnal symptoms and daytime symptoms of insomnia formed separate factors (Pallesen et al., 2008). An exploratory factor analysis in our dataset reproduced the two-factor solution with the first three items loading onto one factor and the three last items loading onto a second factor. We therefore created two parcels for the insomnia items, one containing the first three items and one containing the last three items.

Although the HSCL-25 was originally thought to capture separate anxiety and depression dimensions, later analyses have suggested a variety of different factor structures (Skogen et al., 2017). An initial exploratory factor analysis in our dataset suggested five factors with eigenvalues greater than the average of the initial communalities (i.e., an analog to the eigenvalue-greater-than-one-rule used for principal component analysis; Afifi et al., 2012). However, the first factor was clearly dominant, with an eigenvalue more than six times greater than the eigenvalues of the other four factors. Moreover, the remaining four factors had eigenvalues that were only marginally larger than the average of the eigenvalues from simulated data (parallel analysis with 10,000 replications). We therefore decided to extract a single factor and then drop the items with high uniqueness (>0.70). The remaining 11 items were combined into three parcels, two with four items each and one with three items.

#### Measurement Invariance Across Samples and Time

Testing for measurement invariance across samples entails several steps, each with successively more restrictions placed on the models. First, we performed a test of configural equivalence, wherein equal factor structures are tested. This is achieved by specifying the same pattern of fixed and free factor loadings in both the frigate and the minehunter sample in a multi-group CFA, and aims at examining whether the GHQ-12 evokes the same cognitive frame of reference for respondents across samples. This model also serves as a baseline model with which later, more restricted models can be compared. Second, we performed a test of measurement invariance, in which factor loadings for like items are constrained to equality across the two samples. This examines whether the associations between like items and the underlying constructs are the same across groups, and thus whether the construct indicators (i.e., the items) are calibrated to the underlying construct in the same manner.

All error variances were allowed to vary freely across the two samples, because the requirement that error variances be equal between groups is considered excessively stringent and of little practical value (Byrne and Watkins, 2003). Because the objective of the current study was not to compare latent factor scores across samples, we also did not constrain intercepts to equality across samples.

The process of testing invariance over time is similar to testing invariance across samples, except that we no longer estimate a multi-group CFA, but instead fit a single model in the frigates sample. For the test of configural invariance, the same number of latent factors are specified at both time-points, with the same pattern of fixed and free factor loadings at each appropriate time-point. In addition, covariance between the corresponding factors at T1 and T2, as well as between residuals for like items, are included to allow for them likely correlating over time. Except of any constraints needed for identification purposes, no other constraints on the factor loadings are included at this time.

As before, the test for measurement invariance involves constraining all factor loadings to be equivalent across time-points. For both invariance across time and samples, the more restricted measurement invariance model is nested in the baseline model that allows all parameters to vary freely and can therefore be statistically compared using a likelihood-ratio test (LR χ^2^).

## Results

Table 1 presents the fit statistics for the different planned models. The unidimensional model (Model 1) as originally proposed showed the worst fit of all models tested (CFI = 0.754, SRMR = 0.079, RMSEA = 0.110, and 90% CI for RMSEA = 0.101 –0.120). The fit statistics improved somewhat with the two multidimensional alternatives without method effects. However, only the three-factor model (Model 3) obtained acceptable statistics on both the SRMR and the RMSEA (SRMR = 0.57, RMSEA = 0.074, and 90% CI for RMSEA = 0.064 –0.085). In Model 2, the two factors correlated *r* = 0.62, whereas in Model 3 the factor correlations were: *r*_(__P_,_N__1__)_ = 0.69; *r*_(__P_,_N__2__)_ = 0.50; and *r*_(__N__1_,_N__2__)_ = 0.81. It should be noted that the factor loading for item P5 (“Been able to face problems”) was non-significant in all models so far. We nevertheless chose not to re-run our models with this item deleted so that our models are as comparable as possible to the models previously tested in the literature.

**TABLE 1:** Fit statistics for the tested models of the 12-item General Health Questionnaire (GHQ-12), *N* = 562.

						RMSEA 90% CI			
Models	χ^2^	*df*	CFI	SRMR	RMSEA	LB	UB	AIC	BIC
Model 1: Unidimensional	423.738***	54	0.754	0.079	0.110	0.101	0.120	9210.885	9366.819
Model 2: 2 correlated factors	264.676***	53	0.859	0.062	0.084	0.074	0.095	9053.823	9214.088
Model 3: 3 correlated factors	207.873***	51	0.896	0.057	0.074	0.064	0.085	9001.020	9169.948
Model 4: Artifactual	227.977***	48	0.880	0.059	0.082	0.071	0.093	9027.123	9209.046
Model 5: Bifactor	165.426***	42	0.918	0.051	0.072	0.061	0.084	8976.572	9184.484

AIC, Akaike information criterion; BIC, Bayesian information criterion; CFI, comparative fit index; CI, confidence interval; LB, Lower Bound; RMSEA, root mean square error of approximation; SRMR, standardized root mean square residual; UB, Upper Bound. ***p < 0.001.

The model with an artifactual factor containing all the negative items (Model 4) did not fit the data better than the three-factor model (CFI = 0.880, SRMR = 0.059, RMSEA = 0.082, and 90% CI for RMSEA = 0.071 – 0.093). In addition, both the AIC and the BIC were smaller for the three-factor model than for the artifactual model. The bifactor model (Model 5), on the contrary, obtained acceptable values on all fit statistics, and had the lowest AIC and BIC values of all models tested (see Table 1).

The standardized factor loadings for the bifactor model are presented in Table 2. Worth noticing first is item P5 that does not load significantly on any of the factors. This item therefore does not seem to be a god marker for either the general factor or the specific factor. In total, the general factor accounts for about 55% of the common variance in the 12 GHQ items (ECV = 0.547).

**TABLE 2:** Factor loadings and variance composition for the bifactor model of the General Health Questionnaire (GHQ-12).

	General factor	Specific factor 1	Specific factor 2
	
	λ	λ	λ
P1 Able to concentrate	0.59	0.05_ns_	
P2 Felt playing useful part in things	0.31	0.62	
P3 Felt capable of making decisions	0.13_ns_	0.53	
P4 Able to enjoy day-to-day activities	0.62	0.22	
P5 Been able to face problems	0.03_ns_	0.02_ns_	
P6 Been feeling reasonably happy	0.63	0.21	
N1 Lost sleep over worry	0.47		0.22
N2 Felt constantly under strain	0.28		0.16
N3 Felt couldn’t overcome difficulties	0.40		0.26
N4 Been feeling unhappy and depressed	0.60		0.38
N5 Been losing confidence in self	0.44		0.74
N6 Been thinking of self as worthless	0.39		0.63
ECV	0.547	0.173	0.280
ω	0.810		
ω_s_		0.668	0.774
ω_h_	0.598		
ω_hs_		0.225	0.360

*ECV, explained common variance; ω, omega; ω_h_, omega hierarchical; ω_hs_, omega hierarchical subscale; ω_s_, omega subscale. Specific factor 1 = The Anxiety/Depression factor containing all positively phrased items. Specific factor 2 = Social dysfunction factor containing all negatively phrased items.*

The omega (ω) value for the general factor is an expression of the amount of observed score variance accounted for by all the constructs that underlie a scale score (Brunner et al., 2012), that is, the general factor and the two specific factors in this instance. Thus, if a unit-weighted total scale score of the 12 GHQ items was created, 81% of the variance in this scale score would be accounted for by the general factor and the two specific factors in combination (ω = 0.81). Omega hierarchical (ω_h_), on the other hand, is an expression of the total amount of observed score variance that is attributable to just the general factor. From Table 2, one can see that approximately 60% of the total score variance is accounted for by the general factor (ω_h_ = 0.598). By taking the square root of ω_h_, we can also get an expression of the correlation between the unit-weighted composite score and the target factor. Thus, the ω_h_ of 0.598 would indicate a correlation of 0.77 between the general factor and the observed total score. Reise et al. (2013a) have suggested that ω_h_ values > 0.50 can be useful in determining whether a composite score provides unique, reliable variance. Conversely, values below this render a composite score based on the indicators very difficult to interpret, as less than half of the observed variance in the composite score would be due to the construct of interest (Gignac and Watkins, 2013).

The omega hierarchical subscale (ω_hs_) is the omega counterpart to ω_h_ applicable to the specific factors. About 23% of the variance in the positive subscale score is accounted for by the specific factor (ω_hs_ = 0.225) and about 36% of the variance in the negative subscale is accounted for by the specific factor (ω_hs_ = 0.360) after controlling for the effects of the general factor. The ω_hs_ values for both the specific factors are quite low relative to their respective omega values (ω_s_ in Table 2), suggesting that much of the reliable variance of the subscale scores can be attributable to the general factor rather than what is unique for these two specific factors (Rodriguez et al., 2016). Dividing the ω_hs_ value by the ω_s_ value gives the relative omega, which shows that only about 34% of the variance in the positive subscale (0.668/0.225) and 46% of the variance in the negative subscale (0.774/0.360) is independent of the general factor.

### Predictive Utility

To test the predictive utility of the general factor versus the two specific factors, we performed structural equation modeling with BIS and HSCL as endogenous latent variables predicted by the general and the two specific factors. Prior to performing the full SEM analysis, we first performed a CFA to verify the measurement portion of the models involving the latent BIS and HSCL factors. This two-factor CFA model resulted in a good fit to the data (CFI = 0.998, SRMR = 0.016, RMSEA = 0.033, and 90% CI for RMSEA = 0.000 – 0.076).

**FIGURE 2:**
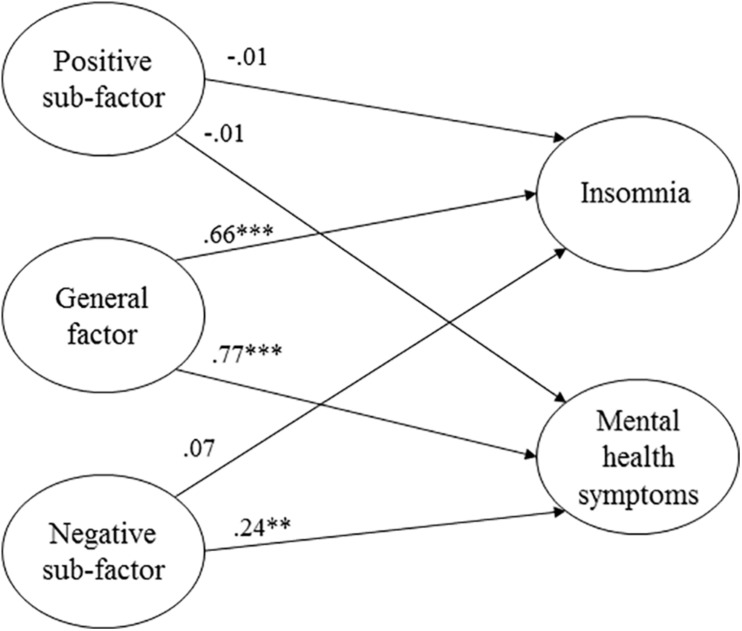
Structural model with the general GHQ-factor and two specific sub-factors predicting symptoms of insomnia and mental health (χ^2^ = 368.737, *p* < 0.001, CFI = 0.92, SRMR = 0.048, RMSEA = 0.069, 90% confidence interval for RMSEA = 0.061 – 0.077). Regression weights are standardized coefficients. ***p < 0.001. **p < 0.01.

The results from the structural model are illustrate in Figure 2. The general factor was strongly and statistically significantly associated with both the BIS and HSCL factors in the expected direction. That is, higher levels on the general GHQ-factor was associated with higher levels of insomnia as measured by BIS and mental health symptoms as measured by HSCL. No associations were found between the positive sub-factor and either BIS or HSCL, whereas the negative sub-factor was positively and statistically significantly associated with mental health symptoms.

### Model Invariance

#### Invariance Across Samples

We started by testing for configural invariance across the frigate and minehunter samples. This entails fitting the same bifactor model structure with all parameters estimated freely across the samples in a multigroup model. This model also serves as a baseline model with which later, more restricted models can be compared. The multigroup bifactor model fit the data acceptably well, χ^2^ = 262.380, *p* < 0.001, RMSEA = 0.075, 90% CI for RMSEA = 0.065 – 0.086, RMSEA = 0.060, and CFI = 0.911.

Next, we constrained all factor loadings to equality in a test of metric invariance. The more restricted metric invariance model is nested in the baseline model that allows all parameters to vary freely and can therefore be statistically compared using a likelihood-ratio test (LR χ^2^). The result of this comparison showed that the model constraining all factor loadings fit the data equally well as the less restricted baseline model, LR χ^2^ = 31.16, *df* = 24, *p* = 0.15.

In sum, these analyses point toward the evidence of equal-form invariance, in that the number of factors and the pattern of factor-indicator relationships are equivalent across samples (i.e., configural invariance). Further, results from the test of metric equivalence suggests that each item contributed to the latent factors to a similar degree across the two samples.

#### Invariance Across Time

Approximately half of the participants from our frigate sample completed the GHQ-12 a second time approximately 6 months after the first administration (*n* = 276). To examine the stability of the bifactor model over time, we next tested for measurement invariance across these two time-points in this sub-sample. Because of missing values on one or more of the GHQ items at either time-points, our sample was further reduced *n* = 248. As with the test of invariance across samples, we started by establishing whether the pattern of loadings was similar across time (i.e., configural invariance). To achieve this, we fitted a model with six factors (two general factors and four specific factors) where all the latent factors loaded on the items for the appropriate time-point. We included correlations between the corresponding factors to allow for the constructs likely being correlated over time. The covariance between non-corresponding factors (e.g., the general factor at T1 and specific factors at T2) was constrained to zero. We also included correlations between the residuals for corresponding items across the time-points to allow for systematic unique variance in the items across time.

The fit of this model was acceptable judged by two of the fit statistics (RMSEA = 0.062 and SRMR = 0.060), but not the third, CFI = 0.879. The general factor correlated *r* = 0.50 over time, whereas the correlations for the specific factors was *r* = 0.26 for the positive factor and *r* = 0.79 for the negative factor. Constraining all factor loadings to be equal across time in a test of metric invariance resulted in a significantly worse fitting model, LR χ^2^ = 50.39, *df* = 24, *p* < 0.01. Inspecting the factor loadings from the initial unconstrained model did suggest discrepancies in some of the factor loadings across the two time-points. Most of these discrepancies were minor, except for one loading on the specific positive factor (item P2) and one loading on the specific negative factor (item N6). Allowing these two items to vary freely across time-points resulted in a non-significant likelihood ratio, LR χ^2^ = 30.94, *df* = 22, *p* = 0.097.

## Discussion

This paper tested a series of alternative factor structures for the widely used GHQ-12 scale. Among the five alternative models tested, a bifactor structure with one general factor and two specific factors proved to be the best representation of the data from a statistical perspective. This model allowed for factor-specific residual variations beyond a general distress factor common to all 12 items. Because these factor-specific variations reflect the different phrasing of the items, with one factor containing entirely negatively worded items and one factor containing entirely positive worded items, they are most likely an expression of method-specific variance (Hankins, 2008; Ye, 2009). Our results therefore suggest that the GHQ-12 is not strictly unidimensional, but rather reflects some multidimensionality due to wording effects. This multidimensionality can pose a challenge when using the GHQ-12 to compute a global distress score by either averaging or summing all items as is commonly done, because this composite may reflect the influence of different sources beyond the general distress factor. In contrast, it is not uncommon for factor analytic studies of psychological measures to reveal minor secondary dimensions in addition to a dominant general factor (Marsh, 1996). In our analyses, the general factor accounted for nearly 60% of the total score variance (ω_h_ = 0.598), while the variance associated with our two specific sub-factors were in contrast relatively small (23 and 36% for the positive and negative factors, respectively). In fact, a larger proportion of the variance associated with the specific factors could be attributed to the general factor than to what was unique to these two factors. As noted by Rodriguez et al. (2016, p. 225), interpreting such factors “as representing the precise measurement of some latent variable that is unique or different from the general factor, clearly, is misguided.” From an applied perspective, we therefore believe that the possible bias introduced to a global composite score due to multidimensionality or wording effects most likely will be small.

That the GHQ-12 items primarily reflect a general factor despite the evidence of some multidimensionality also implies that creating sub-factor or subscale scores is most likely of limited usefulness. This was also illustrated in our SEM analysis, where the general factor had strong associations with BIS and HSCL, whereas the two specific factors were non-significantly or considerably weaker associated with the criterion variables. Other researchers who have assessed the predictive validity of subscales vis-à-vis a general factor have reached similar conclusions. For instance, both Gao et al. (2004) and Shevlin and Adamson (2005) found the three-factor solution based on Graetz (1991) to be the best representation of the data. However, when examining the utility of the three subscales, they appeared to provide little information beyond that of a general factor. Gao et al. (2004) also conclude that there is little need to consider the multidimensionality, but rather that “from a pragmatic point of view we consider it acceptable to use this instrument as a one-dimensional measure” (p. 6).

The final finding from our study is that the bifactor model proved to be fairly robust across different samples. We found the structure to be invariant across two different military samples, one comprising crewmembers from frigates and the other comprising crewmembers from minehunters/sweepers. Our results show that the participants in the two samples responded to the items in a similar manner and attributed the same meaning to the latent factors. In contrast, the bifactor model was less robust when tested for invariance across two time-points in the frigate sample. Our baseline model that specified the same pattern of fixed and free loadings at the two time-points did not provide a good fit to the data, at least as judged by one of the fit statistics we used (CFI = 0.879). This suggest that the crewmembers on board the frigates did not conceptualize the constructs in the same way at the two different time-points. The prerequisite for further testing the metric invariance across time was therefore strictly speaking not met.

One reason for the change in conceptualization of the items could be due to an end-effect of the missions. End-effects represent a change in evaluations and performance at the end of a task, and a prerequisite for such an effect is the knowledge of the endpoint of the task. Since the post-evaluation of the crew was performed in transit back to home base, all crewmembers had a knowledge of the termination of the mission and the evaluation was performed close to this endpoint. End-effects have been found in several domains of psychology (e.g., Catalano, 1973; Lai, 2008). Using GHQ as a measure of mental well-being, Taylor (2006) found a positive end-effect with an increased well-being at late compared to data collected early in the week.

### Limitations

Our analyses were limited to the Likert scoring system of the GHQ-12. While this is a popular approach, other methods have been proposed and used in the literature (for an overview, see Rey et al., 2014). One option is to dichotomize the items by collapsing the first two response categories and the last two response categories and scoring them as respectively “0” and “1” (GHQ-0011). A slightly different approach is to use the above scoring system for the positive items but collapse the last three response categories (0-1-1-1) for the negative items. Finally, different Likert-type formats have also been used, such as a six-point (Kalliath et al., 2004) or a seven-point scale (Ye, 2009). Obviously, our results do not extend to these different scorings systems. On the contrary, there is evidence suggesting that the scoring system can affect the number of factors as well as the particular pattern of item-factor loadings (Aguado et al., 2012; Gao et al., 2012; Rey et al., 2014).

The military samples used in the present study must be considered when assessing the generalizability of our results to other samples and settings. It is conceivable that military personnel may differ from other occupational groups and/or the general population in how they perceive and respond to the GHQ-12. As far as we know, there is limited research that have compared or tested differences in GHQ-12 between military samples and other occupational groups. One exception is a study by Gouveia et al. (2012) that found some minor differences in the factor structure between a military group and the other groups included (students, schoolteachers and the general population). However, the authors conducted no direct statistical comparisons of the different samples.

It must, however, also be stressed that the Norwegian Armed Forces is based on mandatory military service for both men and women, and thus the crew onboard Norwegian naval vessels consist both of professional soldiers, mainly officers and non-commissioned officers, as well as lower rank mandatory conscripts. One argument behind conscription for both women and men is to secure a better cross-section of the population. Selection procedures do of course introduce some limitations regarding who is allowed to serve, as psychopathology and subclinical and clinical symptoms of adjustment disorders are exclusion criteria. In our view this could be said to work to our advantage as the GHQ is not intended for severe pathology and such cases could introduce unwanted noise to our data.

Although we acknowledge that the generalizability of our sample constitutes a limitation, we would like to stress that there are also advantages associated with using military samples. Most importantly, naval vessels are relatively isolated units. This entails that the personnel onboard is exposed to approximately the same levels of isolation from significant others, the same environmental influences, the same types of stressors like exercises, and so on. Compared with civilian samples, our naval samples thus offer greater control over external factors that can produce symptoms of psychological distress and ensures that everyone onboard is exposed to roughly the same types and levels.

## Conclusion

Overall, our results are congruent with the suggestion of Hankins (2008) and Ye (2009), and serval others that item wording can introduce response bias to the GHQ-12. As a result, the multidimensionality demonstrated in many previous studies can be an expression of method effects, specifically, the division of GHQ-12 into positively and negatively phrased items. As such, the GHQ-12 is not strictly unidimensional, but in addition contains factor-specific variations associated with the items wording. However, the explained unique variance associated with these specific factors was relatively small. The consequences of ignoring this multidimensionality and instead use a composite score are therefore most likely small for most practical purposes.

## Data Availability Statement

The raw data supporting the conclusions of this article will be made available by the authors, without undue reservation, to any qualified researcher.

## Ethics Statement

Ethical review and approval was not required for the study on human participants in accordance with the local legislation and institutional requirements. The patients/participants provided their written informed consent to participate in this study.

## Author Contributions

BJ organized the data collection. SH and BJ contributed to the theory development, design and writing of the manuscript. SH primarily conducted the statistical analyses.

## Conflict of Interest

The authors declare that the research was conducted in the absence of any commercial or financial relationships that could be construed as a potential conflict of interest.
